# Soil Fauna Affects Dissolved Carbon and Nitrogen in Foliar Litter in Alpine Forest and Alpine Meadow

**DOI:** 10.1371/journal.pone.0139099

**Published:** 2015-09-25

**Authors:** Shu Liao, Wanqin Yang, Yu Tan, Yan Peng, Jun Li, Bo Tan, Fuzhong Wu

**Affiliations:** 1 Long-term Research Station of Alpine Forest Ecosystems, Key Laboratory of Ecological Forestry Engineering in the Upper Reaches of the Yangtze River of Sichuan Province, Institute of Ecology and Forestry, Sichuan Agricultural University, Chengdu, China; 2 Collaborative Innovation Center of Ecological Security in the Upper Reaches of the Yangtze River, Chengdu, China; Chinese Academy of Sciences, CHINA

## Abstract

Dissolved organic carbon (DOC) and total dissolved nitrogen (TDN) are generally considered important active biogeochemical pools of total carbon and nitrogen. Many studies have documented the contributions of soil fauna to litter decomposition, but the effects of the soil fauna on labile substances (i.e., DOC and TDN) in litter during early decomposition are not completely clear. Therefore, a field litterbag experiment was carried out from 13th November 2013 to 23rd October 2014 in an alpine forest and an alpine meadow located on the eastern Tibetan Plateau. Litterbags with different mesh sizes were used to provide access to or prohibit the access of the soil fauna, and the concentrations of DOC and TDN in the foliar litter were measured during the winter (the onset of freezing, deep freezing and thawing stage) and the growing season (early and late). After one year of field incubation, the concentration of DOC in the litter significantly decreased, whereas the TDN concentration in the litter increased. Similar dynamic patterns were detected under the effects of the soil fauna on both DOC and TDN in the litter between the alpine forest and the alpine meadow. The soil fauna showed greater positive effects on decreasing DOC concentration in the litter in the winter than in the growing season. In contrast, the dynamics of TND in the litter were related to seasonal changes in environmental factors, rather than the soil fauna. In addition, the soil fauna promoted a decrease in litter DOC/TDN ratio in both the alpine forest and the alpine meadow throughout the first year of decomposition, except for in the late growing season. These results suggest that the soil fauna can promote decreases in DOC and TDN concentrations in litter, contributing to early litter decomposition in these cold biomes.

## Introduction

Dissolved organic carbon (DOC) and total dissolved nitrogen (TDN) are often considered as important active biogeochemical pools in carbon and nitrogen cycles [1,2]. The DOC and TDN lost from litter represent a key way in which carbon and nitrogen are transported from plant to the soil [3] and constitute the majority of the available energy and nutrient inputs for the soil fauna and other organisms [1,2]. Conversely, in concert with other decomposers, soil fauna may alter the dynamic patterns of DOC and TDN in litter through feeding, fragmentation and controlling the activity of microorganisms [4–7], thus contributing to litter decomposition and related material cycling [8]. Soil fauna-induced increases in the loss rates of DOC and TDN could greatly affect the present and future labile pools and cycling of carbon and nitrogen. However, little information is available on the effects of soil fauna on the two labile compounds.

Many researchers have considered winter to be a dormant period when the activity of the soil fauna ceases, and the decomposition of litter slows [9,10]. However, recent studies have shown that some soil fauna can adapt to low temperatures and other harsh environmental conditions in the winter [11–14]. These active soil fauna contribute greatly to litter decomposition [15,16]. The majority of foliar litter often falls before the winter, and this fresh litter always contains rich dissolved substances. The gut processing of litter material by soil fauna may directly accelerate the initial loss of these labile substances. In addition, the increased litter surface area and associated physically fragmented substrate resulting from the action of the soil fauna may indirectly promote the loss of DOC and TDN in litter. Moreover, the soil fauna can substantially alter the physical and chemical properties of soil as well the functional structure and activity of microbial communities and, thus, indirectly reinforce the process of litter decomposition [7,16,17]. However, the activity of soil fauna is sensitive to several biotic and abiotic factors. Many reviews have shown that warm and wet conditions can promote fauna activity [16,18], but frequent freeze-thaw events induce changes in the functional structure of the soil fauna in cold biomes [19]. In addition, the varying palatability of litter due to different substrate quality directly affects the activity of soil fauna with regard to litter decomposition [15]. These physical and chemical conditions may alter the soil fauna-induced dynamic patterns of DOC and TDN during litter decomposition; however, such influences are not completely understood. Thus, a field litterbag experiment was conducted to assess the effects of the soil fauna on litter DOC and TDN dynamics during early decomposition according to the hypothesis that the soil fauna will accelerate the decreases in DOC and TDN concentrations in foliar litter in cold biomes.

The alpine forest and the alpine meadow on the eastern Tibetan Plateau are representative cold ecosystems, which are sensitive to climate change [20]. The freezing and thawing period in the winter and early spring stretches over approximately half of the year [21]. The soil is shallow due to frequent geological disasters and low temperature, and the nutrients produced through litter decomposition therefore greatly contribute to both the formation of soil and aboveground productivity [22]. Some studies have found that the soil fauna is a major force in promoting litter decomposition [23,24], but the effect of the soil fauna in cold biomes is not clear. To clarify how the soil fauna affects dissolved substances, we conducted a one-year field experiment at two contrasting sites (alpine forest and alpine meadow) and measured the DOC and TDN concentrations in the foliar litter at various stages as litter decomposition proceeded. Litterbags with different mesh sizes were used to provide or prohibit the access of the soil fauna. The results are expected to enrich our understanding of the litter decomposition process with additional details and may also provide information for predicting current and future ecosystem carbon and nitrogen cycling.

## Material and Methods

### Ethics Statement

All researches involved in our study were in compliance with laws in the People’s Republic of China, and the permission for field experiment in the Miyaluo Nature Reserve was obtained from the Forestry Bureau of Western Sichuan of China. Our field work had negligible effect on environmental pollution and no measurements on humans, animals and endangered plants were involved.

### Site description

This study was conducted in an alpine forest (31°14’ N, 102°53’ E, 3582 m *a*.*s*.*l*.) and an alpine meadow (31°85’ N, 102°68’ E, 3989 m *a*.*s*.*l*.), which are both located on the eastern Tibetan Plateau. The mean annual temperature and precipitation in the alpine forest are 2.7°C and 850 mm, respectively. The dominant species in the alpine forest are cypress (*Sabina saltuaria*) in the canopy and willow (*Salix paraplesia*) in the shrub layer (Table 1). The mean annual temperature and precipitation in the alpine meadow are 3°C and 600 mm, respectively. Wormwood (*Ajania nubigena*) and sedge (*Carex atrofusca*) dominate the alpine meadow (Table 1). Snow begins to accumulate in late October and melts in April of the following year with a highest snow depth of approximately 50 cm in both the alpine forest and the alpine meadow. The soils are classified as Cambisol (World Reference Base taxonomy) [25] in both sites. The pH, total nitrogen and total phosphorus of soil organic layer are 4.76, 28.73 g kg^-1^ and 0.14 g kg^-1^ in the alpine forest, 5.02, 48.50 g kg^-1^ and 0.09 g kg^-1^ in the alpine meadow, respectively.

**Table 1:** Initial concentration of total carbon (TC), total nitrogen (TN), total phosphorus (TP), cellulose and lignin of four litter types (mean ± SE, *n* = 3).

Species	TC (g kg^-1^)	TN (g kg^-1^)	TP (g kg^-1^)	Lignin (%)
*S*. *saltuaria*	462.99 ± 14.99^a^	20.85 ± 1.02^b^	1.73 ± 0.03^b^	28.10 ± 2.49^ab^
*S*. *paraplesia*	337.59 ± 0.71^c^	26.69 ± 1.88^a^	1.94 ± 0.03^a^	24.80 ± 1.20^ab^
*A*. *nubigena*	389.98 ± 2.92^b^	24.30 ± 0.10^ab^	1.52 ± 0.03^c^	21.47 ± 0.98^b^
*C*. *atrofusca*	353.78 ± 1.78^c^	23.59 ± 0.17^ab^	1.48 ± 0.05^c^	29.41 ± 1.27^a^

Different lowercase letters indicate significant differences among species (*p*<0.05).

### Experimental design

**Fig 1:**
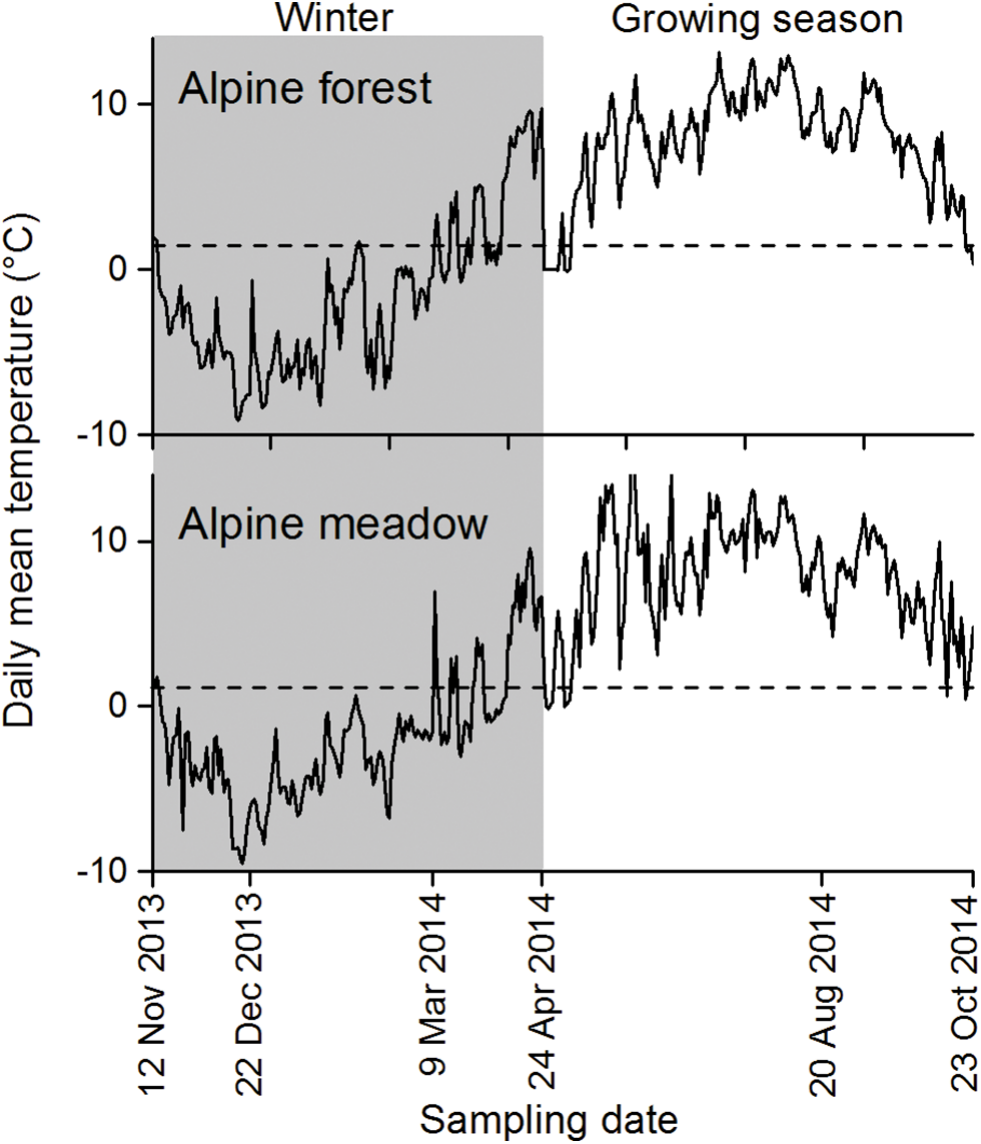
Daily mean temperature in the alpine forest and the alpine meadow during the one-year decomposition experiment. Wintertime is shaded gray.

In October 2013, foliar litter of cypress, willow, wormwood and sedge were collected from around the sampling sites and then air-dried for two weeks. Samples weighing 10 g were placed in nylon litterbags (20 cm × 20 cm in size) with mesh sizes of 0.04 mm on the bottom and 0.04 mm (to exclude soil fauna) or 3.00 mm (to permit soil fauna) on the top [26,27]. On 12nd November 2013, 30 litterbags (3 replicates × 2 treatments × 5 sampling dates) filled with litter of each species were placed on the soil surface at their original site of collection. Three litterbags were harvested in each plot on 22nd December 2013, 9th March, 24th April, 20th August and 24th October 2014. These sampling dates denote the approximate ends of the onset of freezing, deep freezing and thawing during winter and the early and late growing season, respectively, at the study area according to our previous long-term observations [28–30]. The air-dried litter was then shattered with a disintegrator, and the powdered subsamples were used to determine moisture contents (through the conversion of air-dried mass to dry mass) and DOC and TDN concentrations. The temperatures of the air and the litterbags were recorded every two hours with data loggers (iButton DS1923-F5, Maxim/Dallas Semiconductor, Sunnyvale, CA, USA) (Table 2; Fig 1).

**Table 2:** The mean temperature (MT), positive (PAT) and negative accumulated temperature (NAT), and number of freeze-thaw cycles (NFTC) in the air and litterbags during the various stages in the alpine forest and the alpine meadow.

			MT(°C)	PAT(°C)	NAT(°C)	NFTC
Onset of freezing stage	Alpine forest	Air	-2.13	23	-110	—
		Litter	-3.68	28	-178	39
	Alpine meadow	Air	-2.74	18	-130	—
		Litter	-3.10	42	-169	65
Deep freezing stage	Alpine forest	Air	-4.58	28	-381	—
		Litter	-3.08	36	-273	42
	Alpine meadow	Air	-3.58	23	-298	—
		Litter	-3.45	1	-267	95
Thawing stage	Alpine forest	Air	2.99	157	-20	—
		Litter	3.97	185	-2	23
	Alpine meadow	Air	1.43	112	-46	—
		Litter	2.22	126	-24	69
Early growing season	Alpine forest	Air	8.08	954	-1	—
		Litter	8.08	954	0	2
	Alpine meadow	Air	8.18	967	-1	—
		Litter	8.45	997	0	14
Late growing season	Alpine forest	Air	6.65	426	0	—
		Litter	7.10	454	0	7
	Alpine meadow	Air	6.62	423	0	—
		Litter	6.74	431	0	14

A 0.5 g air-dried powered subsample was transferred to a screw-cap tube that was then filled with 40 ml of deionized water and shaken for 30 minutes, followed by passage through a 0.45 μm filter. DOC and TDN concentrations were analyzed using a TOC analyzer (multi N/C 2100, Analytik Jena, Thüringen, Germany) and the Kjeldahl method (KDN, Top Ltd., Zhejiang, China), respectively. All values were calculated based on the dry mass.

### Statistical analyses

A repeated measures ANOVA with the Greenhouse-Geisser correction using the *F* test, if the Mauchly test was not certified, was performed to evaluate the effects of the litter species and soil fauna on the DOC and TDN concentrations over time. At each stage, a two-way ANOVA was employed to test the effects of the litter species and soil fauna. Levene’s test for homogeneity of variance was performed before conducting the ANOVAs. A pairwise t-test was carried out to assess the significant differences between the soil fauna and no soil fauna treatment at three *P*-levels (* *P*<0.05; ** *P*<0.01; *** *P*<0.001). These analyses were performed using SPSS 20.0 (IBM SPSS Statistics Inc., Chicago, IL, USA).

## Results

### Dissolved organic carbon

**Fig 2:**
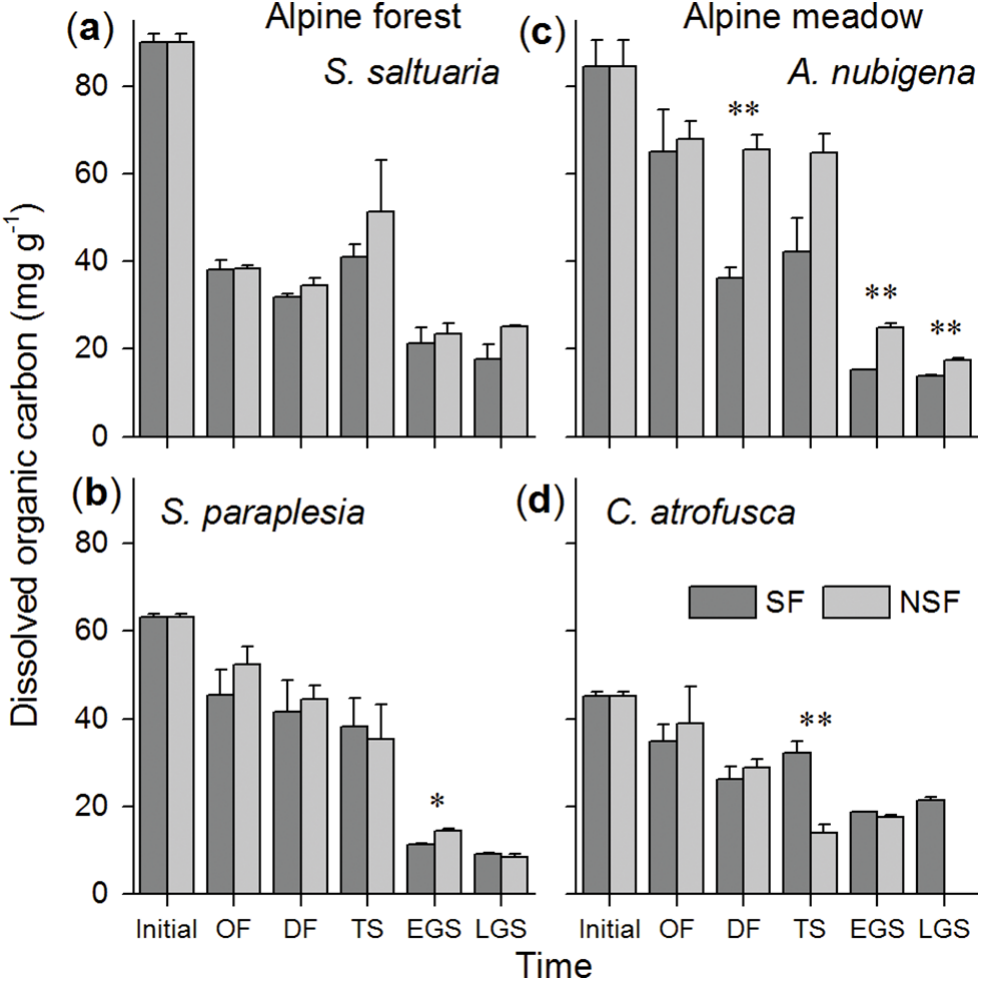
The concentration of dissolved organic carbon in (a) cypress, (b) willow, (c) wormwood and (d) sedge foliar litter in the soil fauna (SF) and no soil fauna (NSF) treatments at different stages in the alpine forest and the alpine meadow. * *P*<0.05 and ** *P*<0.01 tested by pairwise t-test.

The concentration of DOC in the litter substantially declined, regardless of the litter species involved, in both the alpine forest and the alpine meadow. As shown by the results of repeated measures ANOVA, the litter quality (factor “species”) (*F* = 55.2, *P*<0.001; Table 3) and soil fauna (*F* = 16.9, *P*<0.01) significantly affected the DOC concentration throughout the entire year of decomposition. However, two-way ANOVA showed that soil fauna did not affected the DOC concentration at the onset of freezing stage (*F* = 2.4, *P*>0.05; Table 4) and the thawing stage (*F* = 1.3, *P*>0.05), when these crucial stages were examined independently. Over the one-year incubation, the soil fauna significantly decreased DOC concentration of 39% for willow litter in the early growing season (*P*<0.05; pairwise t-test; Fig 2B). The decreases in the DOC concentration in wormwood litter were significantly (*P*<0.01; Fig 2C) promoted by 45%, 39% and 21% in the soil fauna treatment at the deep freezing and the early and late growing season as compared to the no soil fauna treatment, respectively. The soil fauna significantly restrained the decrease in the DOC concentration by 131% in sedge litter at the thawing stage (*P*<0.01; Fig 2D). However, the effects of the soil fauna were not significant (*P*>0.05; Fig 2A) for cypress litter at all stages involved in this study.

**Table 3:** Results of repeated measures ANOVA of time, litter species and soil fauna on the concentrations of dissolved organic carbon (DOC) and total dissolved nitrogen (TDN) as well as the DOC:TDN ratio (DOC/TDN) over time. The interaction terms are nested in parentheses.

Source of variation	*df*	*F*_DOC_	*F*_TDN_	*F*_DOC/TDN_
Time	4	208.0***	51.4***	178.2***
Species	3	55.2***	1750.0***	121.4***
Soil fauna	1	16.9**	18.0**	2.8
Time (Species)	12	20.0***	56.8***	64.1***
Time (Soil fauna)	4	3.0*	0.6	13.2***
Species (Soil fauna)	3	11.8**	11.9***	0.9
Time (Species) (Soil fauna)	12	7.0***	3.7*	15.8***

* *P*<0.05

** *P*<0.01

*** *P*<0.001.

**Table 4:** *F* values of a two-way ANOVA with Tukey’s HSD testing for the concentrations of dissolved organic carbon (DOC) and total dissolved nitrogen (TDN) as well as the DOC:TDN ratio (DOC/TDN) for the effects of the litter species, soil fauna and their interactions at each stage.

Source	*df*	DOC	TDN	DOC/TDN
Onset of freezing stage				
Species	3	36.2***	28.6***	55.5***
Soil fauna	1	2.4	0.0	6.9*
Species × Soil fauna	3	0.4	5.0*	15.7***
Deep freezing stage				
Species	3	55.2***	283.4***	60.2***
Soil fauna	1	15.3***	2.5	20.1***
Species × Soil fauna	3	22.8***	6.6**	8.9**
Thawing stage				
Species	3	24.4***	111.8***	118.6***
Soil fauna	1	1.3	1.6	7.5*
Species × Soil fauna	3	10.9***	1.6	5.2*
Early growing season				
Species	3	40.6***	344.2***	140.2***
Soil fauna	1	28.8***	3.8	13.4**
Species × Soil fauna	3	12.3***	26.4***	1.3
Late growing season				
Species	3	106.0***	11.5**	12.8**
Soil fauna	1	25.1***	0.2	0.2
Species × Soil fauna	3	11.1**	3.2	0.1

* *P*<0.05

** *P*<0.01

*** *P*<0.001.

### Total dissolved nitrogen

**Fig 3:**
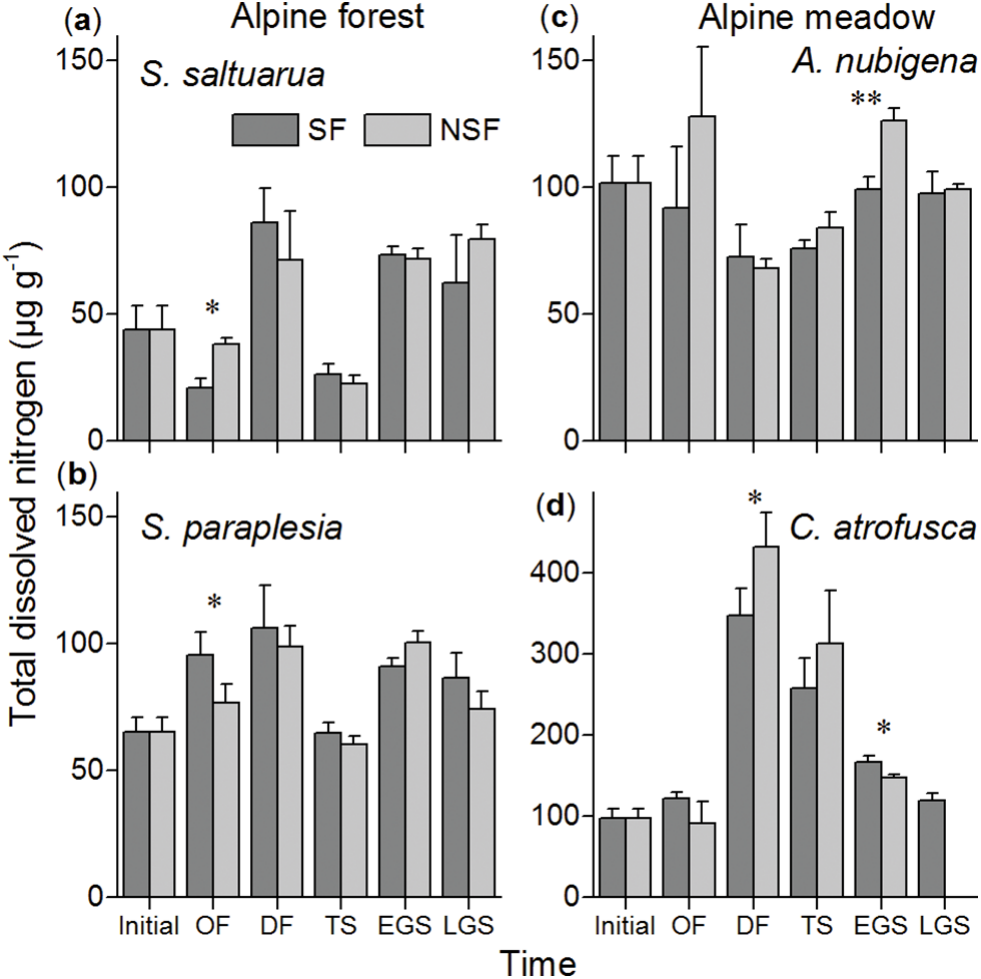
The concentration of total dissolved nitrogen in (a) cypress, (b) willow, (c) wormwood and (d) sedge foliar litter in the soil fauna (SF) and no soil fauna (NSF) treatments at different stages in the alpine forest and the alpine meadow. * *P*<0.05 and ** *P*<0.01 tested by pairwise t-test.

**Fig 4:**
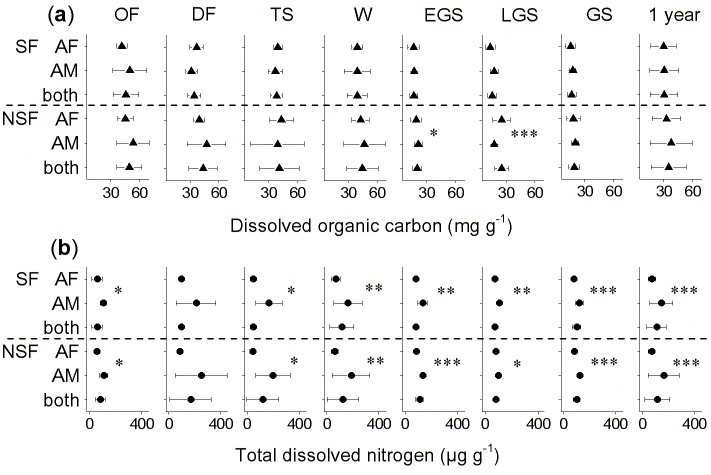
Means and 95% confidence intervals of the (a) dissolved organic carbon and (b) total dissolved nitrogen concentrations after each stage as well as in the winter, the growing season and for the entire year of decomposition in the alpine forest (AF) and the alpine meadow (AM) with the related litter combined, in the soil fauna (SF, above dashed line) and no soil fauna (NSF, below dashed line) treatments. * *P*<0.05, ** *P*<0.01 and *** *P*<0.001 tested by pairwise t-test.

The concentration of TDN in the foliar litter increased during the one-year incubation, except in the wormwood litter. The results of repeated measures ANOVA showed that litter quality (factor “species”) significantly (*F* = 1750.0, *P*<0.001; Table 3) affected the dynamics of the TDN concentration over time. The results of two-way ANOVA showed that the effects of the litter species and soil fauna were significant (*P*<0.001; Table 4) and not significant (*P*>0.05), respectively, at all independent crucial stages. The soil fauna significantly (*P*<0.05; pairwise t-test) decreased TDN concentration by 45% for cypress litter at the onset of freezing stage (Fig 3A). Meanwhile, the soil fauna significantly (*P*<0.01) decreased by 22% and 19% in wormwood litter (Fig 3C) in the early growing season and in sedge litter (*P*<0.05; Fig 3D) at the deep freezing stage, respectively. In contrast, the soil fauna significantly (*P*<0.05; Fig 3B and 3D) constrained the decreases at the onset of freezing stage and in the early growing season for both willow and sedge litter by 25% and 14%, respectively. The dynamics of TDN varied significantly (*P*<0.05; pairwise t-test; Fig 4B) between the alpine forest and the alpine meadow in most crucial stages.

### DOC/TDN ratio

**Fig 5:**
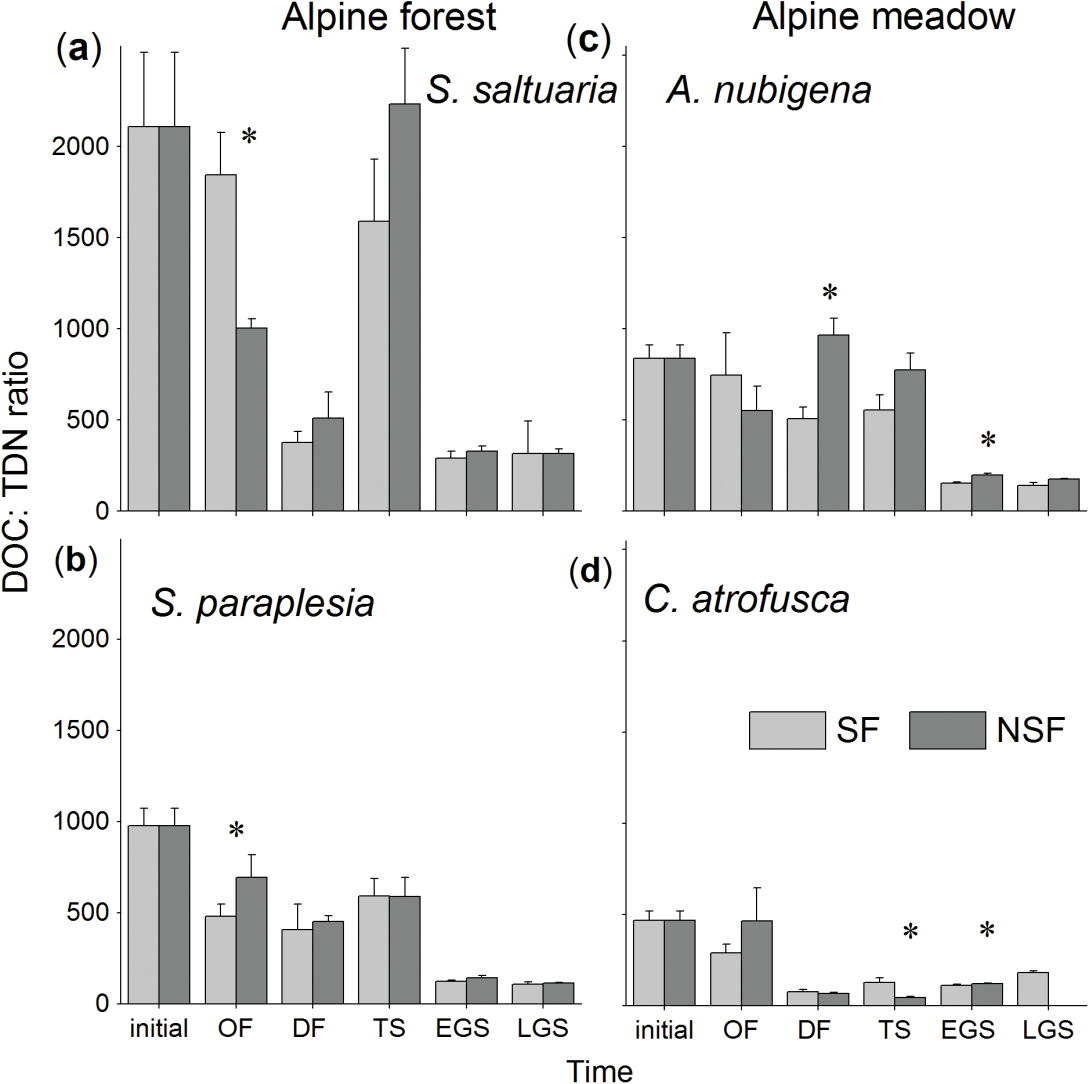
The ratios of dissolved organic carbon and total dissolved nitrogen in (a) cypress, (b) willow, (c) wormwood and (d) sedge foliar litter in the soil fauna (SF) and no soil fauna (NSF) treatments at different stages in the alpine forest and the alpine meadow. * *P*<0.05 tested by pairwise t-test.

The DOC/TDN ratio declined, irrespective of the litter species involved. However, there was a significant increase in the thawing stage for cypress litter compared with the deep freezing stage in both the soil fauna (*P*<0.05, pairwise t-test; Fig 5A) and no soil fauna (*P*<0.01) treatment. The DOC/TDN ratio showed significant (all *P*<0.05) decreases from 2110 to 317, 976 to 106, 837 to 142 and 467 to 179 in cypress, willow, wormwood and sedge litter, respectively (Fig 5A–5D), after one year of decomposition in the soil fauna treatment. In addition, the decreasing pattern of the DOC/TDN ratio observed in the no soil fauna treatment was similar to the consequences of the soil fauna treatment (*P*>0.05; Fig 5A–5D), regardless of the litter species. The results of two-way ANOVA showed that the effects of the soil fauna were significant (*P*<0.05) in altering the ratio, except during the late growing season.

## Discussion

The hypothesis that the soil fauna would accelerate the decreases in DOC and TDN concentrations was supported in the present study. The results presented herein indicated that the soil fauna significantly decreased the concentrations of DOC and TDN in the litter over an entire year of decomposition, although the effects on TDN were much smaller than those on DOC. Thus, our results indicated that the soil fauna has a positive effect on promoting the cycling of carbon and nitrogen and then improving the soil fertility in these cold biomes.

The onset of freezing, deep freezing and thawing stage were regarded as the entire winter in this study [28–30]. Because a number of soil fauna can adapt to low temperatures and frequent freeze-thaw events [12,13], the soil fauna still has a considerable effect on the changes in the DOC and TDN concentrations as well as the DOC/TDN ratio in the winter. However, varying conditions due to snow cover, in addition to freeze-thaw cycles, have different effects on the activity of soil fauna to different extents. First, foliar litter often falls before winter, and fresh litter is assumed to be the most attractive to the soil fauna due to the presence of rich dissolved substances and the high palatability of the litter [15,23]. Nevertheless, the soil fauna only significantly decreased TDN at the onset of freezing stage in cypress and willow litter (Fig 3A and 3B) in the alpine forest, thus leading to an substantial change in the DOC/TDN ratio compared with the no soil fauna treatment for these two litter species (Fig 5A and 5B). This unexpected observation may be explained by the initial low temperature and freezing events that reduced the activity of the soil fauna. However, compared with the alpine meadow, the hydrothermal conditions under canopy trees or the soil layer in the alpine forest may provide safer sites for soil fauna when faced with such constrained ambient environment [12,30,31]. The migration of the soil fauna to more favorable conditions may reduce access to fresh litter. Second, a deep freezing stage was characteristic of the low temperature and frequent freeze-thaw cycles (Table 2), which may cause great physical damage to both the litter material and the soil fauna [32]. In contrast, a thick layer of snow has the ability to insulate and increase warmth [29], which may provide a relatively suitable environment for soil fauna [33], allowing it to survive to some degree. After soil thawing, the litter that was fragmented due to physical fragmentation caused by freezing events may be more accessible to the soil fauna. All of the above situations together may be the reason that the soil fauna still significantly decreased DOC concentration in wormwood litter and in TDN in sedge litter in the deep freezing stage (Figs 2C and 3D). Finally, the effects of the soil fauna may be weakened by widespread thawing events at the end of the winter. Considerable amounts of many soluble substances were lost along with the thawing ice in both the soil fauna and no soil fauna treatments, resulting in almost no difference in the DOC and TDN concentrations or their ratio between the two treatments.

The activity of the soil fauna recovered as temperature increases, a significant increasing abundance of the soil fauna communities was found compared with winter (S1 Table). And thus, the soil fauna significantly decreased DOC concentration in willow (Fig 2B) and wormwood litter (Fig 2C) and in TDN in wormwood litter (Fig 3C) in the growing season. However, under the relatively stable and favorable conditions in the growing season, the differences in chemical qualities between litter species may be the dominant factor determining the various efficiencies of the soil fauna effects during litter decomposition. Litter of a high quality (high nitrogen concentration and low C:N and lignin:N ratios [34]) is usually more palatable for soil fauna [15]. According to our results, the deciduous willow (Fig 2B) and the dicotylous wormwood litter (Fig 2C) showed greater decreases in the DOC concentration in the litter compared with the coniferous cypress litter (Fig 2A) and the monocotylous sedge litter (Fig 2D), respectively, due to presenting higher litter quality (unpublished data). These tendencies are similar to the results of previous studies on litter quality among species [7,35,36].

In addition, the DOC concentration was gradually decreased as litter decomposition proceeding both in the alpine forest and the alpine meadow, and even the growing season was associated with a smaller decrease compared with the winter. A possible reason may be that the carbon comes from fresh litter is one of the major energy source for soil fauna [37], thus soil fauna greatly uptake the carbon in fresh litter. Meanwhile, the loss of dissolved substances due to frequent thawing events during late winter led a very limited DOC remaining in the litter. The lower palatability for soil fauna in the late decomposition stage and thus may relax soil fauna affecting litter decomposition [38]. By contrast, the concentration of TDN has not showed a clear and regular trend but slightly increased after the whole year decomposition compared with the initial value. This may contribute to the relative increase in comparing with other compounds. For example, when nitrogen in litter is not reach the need of decomposers, decomposers will immobilize inorganic nitrogen in surrounding environment, along with continuously decreased carbon, leading an increasing TDN concentration [37]. Furthermore, organic nitrogen could be mineralized to inorganic nitrogen during decomposition [39].

Numerous studies have documented that climate along with climate-induced micro-environmental conditions and litter quality generally affects the soil fauna [7,16,40]. The alpine forest investigated in our study, which is located at an altitude 407 m lower than the alpine meadow, shows more favorable conditions for the soil fauna due to protection from direct sunlight by the canopy [30] and the water holding capacity of its trees, shrub and grass (Table 2). Although soil fauna showed heterogeneous effects on TDN between the alpine forest and the alpine meadow, the differences in TDN concentrations were similar between the two sites both in the soil fauna and no soil fauna treatments (Fig 4B). Thus, we considered that the effects of the soil fauna on DOC and TND concentrations showed no differences between the alpine forest and the alpine meadow. Similar fauna communities were found at both sites according to our previous work [41] (S1 Table), and global warming as well as the increase in warmth caused by snow cover and bryophytes [33,42] may explain this observation. Additionally, the soil fauna may be affected by the expansion of shrubs into the tundra in high latitude regions and at high altitudes [43] and by the decreasing palatability of the litter because of the decreased litter quality induced by global warming [15]. Thus, soil fauna-induced litter decomposition and the cycling of carbon and nitrogen may show a new pattern in the future in cold biomes.

In conclusion, based on a one-year field incubation experiment, we found that the soil fauna promotes the decreases in DOC and TDN concentrations in litter. These positive effects of the soil fauna can promote the process of litter decomposition, and thus the cycling of carbon and nitrogen within the ecosystem and the improvement of local soil fertility. There was no significant or consistent tendency of the effects of the soil fauna on the TDN concentration according to the entire year of decomposition, which indicates that the pattern of the decrease in dissolved nitrogen maybe more dependent on microorganisms or environmental conditions than on the soil fauna in this area. These results suggest that soil fauna can promote the cycling of labile substances in foliar litter during early decomposition in these cold biomes.

## Supporting Information

S1 Dataset The concentrations of dissolved organic carbon and total dissolved nitrogen and DOC/TDN ratios in foliar litters at different stages in the alpine forest and the alpine meadow. (XLSX)

S1 Table Soil fauna communities in the litterbags of soil fauna treatment at different stages during the one-year decomposition. (DOC)
