# Supplementary material for: Soil Fauna Affects Dissolved Carbon and Nitrogen in Foliar Litter in Alpine Forest and Alpine Meadow
Source: PLoS One. 2015 Sep 25;10(9):e0139099. doi: 10.1371/journal.pone.0139099 (PMC4583391; doi:10.1371/journal.pone.0139099)
Supplement: S1 Table — (DOC) [file pone.0139099.s002.doc]

**Supporting information** for

**Soil Fauna Affects Dissolved Carbon and Nitrogen in Foliar Litter in Alpine Forest and Alpine Meadow**

Shu Liao^1,2^, Wanqin Yang^1,2^, Yu Tan^1,2^, Yan Peng^1,2^, Jun Li^1,2^, Bo Tan^1,2^, Fuzhong Wu^1,2*^

**1** Long-term Research Station of Alpine Forest Ecosystems, Key Laboratory of Ecological Forestry Engineering in the Upper Reaches of the Yangtze River of Sichuan Province, Institute of Ecology and Forestry, Sichuan Agricultural University, Chengdu, China, **2** Collaborative Innovation Center of Ecological Security in the Upper Reaches of the Yangtze River, Chengdu, China

* Corresponding author

E-mail: [wufzchina@163.com](mailto:wufzchina@163.com) (FW)

**Table S1** Soil fauna communities in the litterbags of soil fauna treatment at different stages during the one-year decomposition.

| Stage | Cypress | Willow | Wormwood | Sedge |
| --- | --- | --- | --- | --- |
| Onset of freezing stage | *Prostigmata* | *Theraphosidae*  *Isotomidae* | *Homoptera*  *Anystidae*  *Isotomidae* | *Nemata*  *Isotomidae*  *Theraphosidae*  *Podura Linnaeus* |
| Deep freezing stage | *Mesostigmata* | / | *Sejidae*  *Anystidae*  *Laelapidae*  *Galumnidae*  *Oribatulidae* | *Sejidae*  *Anystidae*  *Galumnidae*  *Oribatulidae* |
| Thawing stage | *Oribatida*  *Prostigmata*  *Isotomidae* | *Prostigmata*  *Isotomidae* | *Anystidae*  *Isotomidae* | *Anystidae*  *Galumnidae*  *Isotomidae*  Oribotritiidae |
| Early growing stage | *Isotomidae*  *Oribatida*  *Prostigmata*  *macropyline oribatid mites*  *Podura Linnaeus*  *Hirudisomatidae*  *Carabidae*  *Scolopendrellidae* | *Isotomidae*  *Oribatida*  *Bolitophilidae*  *Prostigmata*  *Scutacaridae*  *Syrphidae*  *macropyline oribatid mites*  *Tarsonmidae*  *Hirudisomatidae*  *Scolopendrellidae* | *Isotomidae*  *Prostigmata*  *Oribatida*  *Enicocephalidae*  *Podura Linnaeus*  *Carabidae*  *Nemata* | *Isotomidae*  *Theraphosidae*  *Oribatida*  *Ditomyiidae*  *Prostigmata*  *Syrphidae*  *Carabidae*  *Podura Linnaeus* |
| Late growing stage | *Isotomidae*  *Prostigmata*  *Oribatida*  *Mycetophilidae*  *Phoridae*  *Entomobryidae*  *Nemata* | *Isotomidae*  *Prostigmata*  *Oribatida*  *Staphylinidae*  *Sminthuridae* | *Isotomidae*  *Prostigmata*  *Homoptera*  *Oribatida*  *Nemata* | *Prostigmata*  *Scydmaenidae*  *Nemata*  *Kalotermitidae*  *Entomobryidae*  *Phlaeothripidae* |
